# Supplementary material for: The causal relationship between the human gut microbiota and pyogenic arthritis: a Mendelian randomization study
Source: Front Cell Infect Microbiol. 2024 Nov 26;14:1452480. doi: 10.3389/fcimb.2024.1452480 (PMC11629706; doi:10.3389/fcimb.2024.1452480)
Supplement: Supplementary file 1 [file DataSheet1.zip › mendelian test/ebi-a-GCST90027700.csv_leave_one_out.pdf]

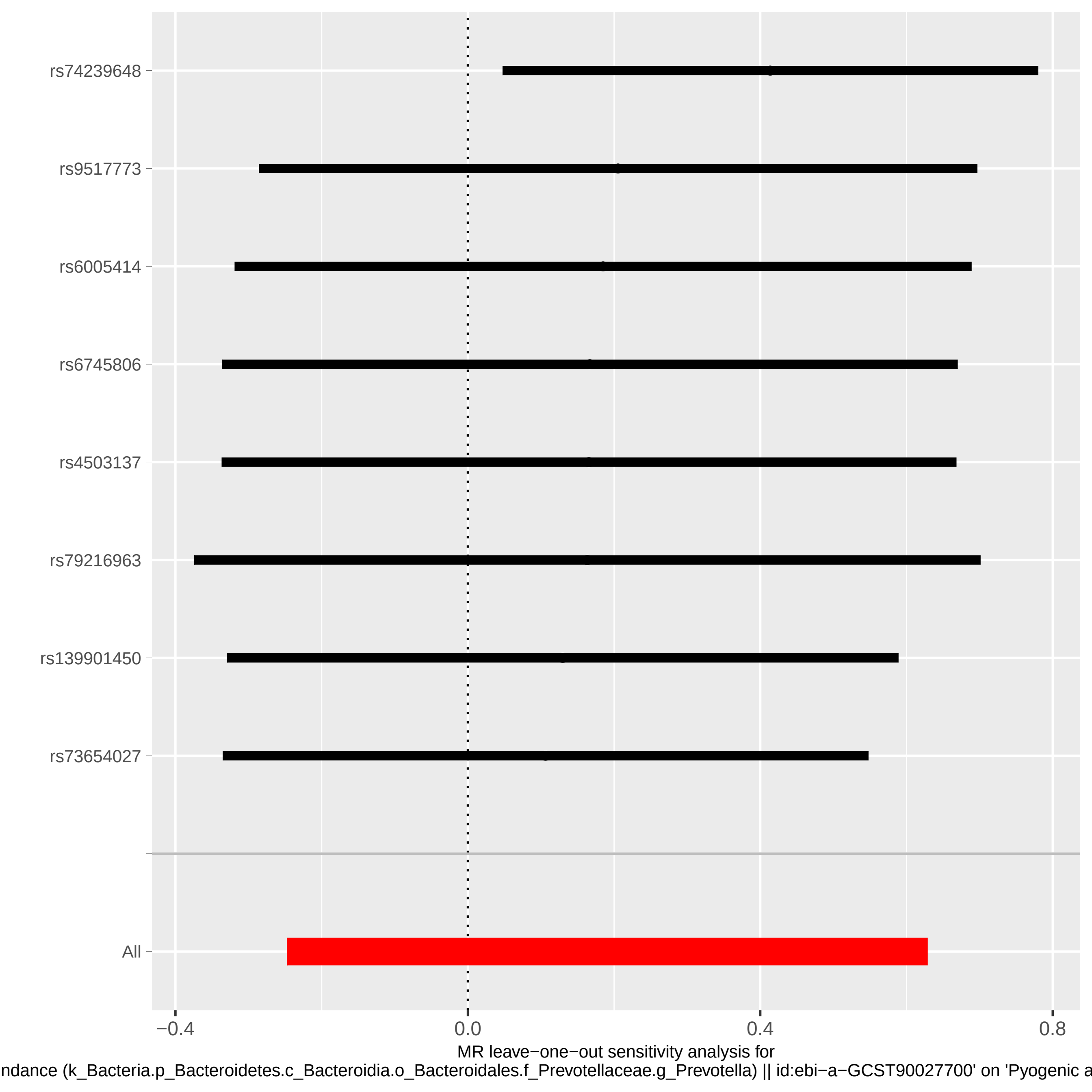
rs74239648
rs9517773
rs6005414
rs6745806
rs4503137
rs79216963
rs139901450
rs73654027
All
−0.4
0.0
0.4
0.8
MR leave−one−out sensitivity analysis for
ndance (k_Bacteria.p_Bacteroidetes.c_Bacteroidia.o_Bacteroidales.f_Prevotellaceae.g_Prevotella) || id:ebi−a−GCST90027700' on 'Pyogenic a
